# IL-36α: a novel cytokine involved in the catabolic and inflammatory response in chondrocytes

**DOI:** 10.1038/srep16674

**Published:** 2015-11-12

**Authors:** Javier Conde, Morena Scotece, Vanessa Abella, Ana Lois, Verónica López, Tomás García-Caballero, Jesús Pino, Juan Jesús Gómez-Reino, Rodolfo Gómez, Francisca Lago, Oreste Gualillo

**Affiliations:** 1 SERGAS (Servizo Galego de Saude) and IDIS (Instituto de Investigación Sanitaria de Santiago), the NEIRID Lab (Neuroendocrine Interactions in Rheumatology and Inflammatory Diseases), Research Laboratory 9, Santiago University Clinical Hospital, Santiago deCompostela, Spain; 2 Department of Molecular and Cellular Biology, University of Coruña (UDC), A Coruña, Spain; 3 Department of Morphologic Sciences, Faculty of Medicine, University of Santiago de Compostela, Santiago deCompostela, Spain; 4 SERGAS (Servizo Galego de Saude), Division of Orthopaedics Surgery and Traumatology, Santiago University Clinical Hospital, Santiago deCompostela, Spain; 5 University of Santiago de Compostela, Department of Medicine and SERGAS (Servizo Galego de Saude) and IDIS (Instituto de Investigación Sanitaria de Santiago), Division of Rheumatology, Santiago University Clinical Hospital, Santiago deCompostela, Spain; 6 SERGAS (Servizo Galego de Saude) and IDIS (Instituto de Investigación Sanitaria de Santiago), cellular and molecular Cardiology Laboratory, Research Laboratory 7, Santiago University Clinical Hospital, Santiago deCompostela, Spain

## Abstract

Recent studies confer to IL-36α pro-inflammatory properties. However, little is known about the expression and function of IL-36α in cartilage. This study sought to analyze the expression of IL-36α in healthy and OA cartilage. Next, we determined the effects of recombinant IL-36α on catabolism and inflammation in chondrocytes. For completeness, part of the signaling pathway elicited by IL-36α was also explored. IL-36α expression was evaluated by immunohistochemistry and RT-qPCR. Expression of MMP-13, NOS2 and COX-2 was also determined in OA articular chondrocytes treated with recombinant IL-36α. IκB-α and P-p38 was explored by western blot. We observed a low constitutive expression of IL-36α in healthy human chondrocytes. However, OA chondrocytes likely expressed more IL-36α than healthy chondrocytes. In addition, immune cells infiltrated into the joint and PBMCs express higher levels of IL-36α in comparison to chondrocytes. OA chondrocytes, treated with IL-36α, showed significant increase in the expression of MMP-13, NOS2 and COX-2. Finally, IL-36α stimulated cells showed NFκB and p38 MAPK activated pathways. IL-36α acts as a pro-inflammatory cytokine at cartilage level, by increasing the expression of markers of inflammation and cartilage catabolism. Like other members of IL-1 family, IL-36α acts through the activation of NFκB and p38 MAPK pathway.

Osteoarthritis (OA) is one of the most common rheumatic disorders and a major cause of pain and disability in older adults. Although, OA is considered a primary disorder of articular cartilage, it is now generally accepted that OA is a disease of the whole joint, and other tissues, including synovia, are also affected^1^.

Chondrocytes, the unique cell type of adult articular cartilage, remain as quiescent cells in normal conditions, maintaining the turnover of the extracellular matrix components. However, during OA, chondrocytes and cells of the synovia, as well as other joint tissues, become activated due to the influence of multiple insults, which include high mechanical stress, degradation products or inflammatory cytokines and adipokines^2^. Chondrocyte activation may result in a phenotypic change, apoptosis and aberrant expression of catabolic and inflammatory-related factors, including nitric oxide, COX-2, ADAMTs or metalloproteinases (MMPs)^2^.

IL-1, the forerunner of a family of cytokines including at present 11 members, is a well-known pro-catabolic factor at cartilage level. It is able to induce the expression of different MMPs, such as MMP-1, MMP-3 and MMP-13 in both cartilage and synovial tissues. In addition, IL-1 increases the synthesis of pro-inflammatory mediators, including nitric oxide, prostaglandin E_2,_ IL-6, and chemokines such as IL-8. All these factors can synergize with one another in promoting and perpetuating chondrocyte catabolic response^3^.

Recently, a new member of the IL-1 cytokine family has been identified, interleukin-36 (IL-36). IL-36 exists as three different forms, IL-36α, β and γ (IL-1F6, IL-1F8, and IL-1F9 respectively)^4^,^5^. IL-36α is a 17 kDa protein able to bind to IL-36 R (formerly named IL-1RL2 or IL-1Rrp2). As other members of the above mentioned family, IL-36α binds to its receptor resulting in the activation of MAPK and nuclear factor-κB (NFκB) pathways^6^.

Several cells have been identified as target of IL-36. Actually, dendritic cells and T lymphocytes responded to IL-36^7^, by increasing the expression of pro-inflammatory cytokines, even more efficiently than other IL-1 family members such as IL-1^7^. Moreover, IL-36 synergizes with IL-12 to promote Th1 polarization of naive T cells^8^. It has been also postulated that IL-36 might play a major role in the development of psoriasis. Indeed, IL-36 transgenic mice showed a psoriasis-like skin phenotype^9^.

Regarding the involvement of IL-36α in joint degenerative diseases, it was demonstrated that this cytokine is highly expressed in synovial tissues from psoriatic arthritic (PsA) and rheumatoid arthritis (RA) patients^10^, suggesting a potential role for IL-36α in the inflammatory response of the synovial tissues. In addition, the β isoform of IL-36 is able to induce the production of several inflammatory mediators in synovial fibroblasts and chondrocytes^11^. Although, several lines of evidence postulated a role for IL-36α in cartilage metabolism, there is no experimental evidence of the activity of this novel cytokine in chondrocytes.

The aim of our study was thus to analyze and to compare the expression of IL-36α in healthy and OA chondrocytes and in other possible cellular sources such as the immune cells migrated into the joint and peripheral blood mononuclear cells (PBMCs). Next, and for the first time, we analyzed the effect of recombinant IL-36α on the expression of different genes involved with degenerative processes of articular cartilage during OA. Finally, we explored the signaling pathway used by IL-36α in OA chondrocytes.

## Methods

For experiments involving humans, all the methods were carried out in accordance with the approved guidelines. All experimental protocols were approved by the local ethics committee (Santiago University Clinical Hospital Ethics Committee (CAEIG 2014/310). Informed consent was obtained from all subjects.

### Reagents

All culture reagents were from Sigma (MO, USA) and Lonza, (Switzerland). For RT-PCR, a First Strand Kit, Master mix, primers for NOS2, COX-2, IL-36R, MMP-13 and GAPDH were purchased from SABiosciences (MD, USA). Nucleospin kits for RNA and protein isolation were from Macherey-Nagel (Germany). Human recombinant IL-36α was from R&D Systems (MN, USA), human recombinant IL-1β was purchased from Immunostep (Salamanca, Spain).

### Cell culture

Human primary chondrocytes culture was developed as previously described^12^,^13^. Briefly, healthy human articular cartilage samples were obtained from joints of patients underwent to joint replacement due to traumatic fractures. Osteoarthritic human cartilage samples were obtained from patients undergoing total joint replacement surgery, with permission from the local ethics committee (Santiago University Clinical Hospital Ethics Committee (CAEIG 2014/310) and informed consent was obtained from all patients participating in the study. Cartilage samples were obtained from the joint area of minimal load with normal morphologic examination (i.e., no change in color and no fibrillation). Human chondrocytes were cultured in DMEM/Ham’s F12 medium supplemented with 10% of fetal bovine serum, L-glutamine, and antibiotics (50 units/ml penicillin and 50 μg/ml streptomycin). Cells were seeded in monolayer up to the high density and used freshly in order to avoid dedifferentiation.

Peripheral blood mononuclear cells (PBMCs) were isolated by Ficoll (GE Healthcare, USA) density-gradient protocol.

Immune cells attached to cartilage were isolated during the chondrocytes primary culture. After cartilage digestion and culture, the present immune cells were recollected and mRNA was extracted.

For RT-PCR and western blot, cells were seeded in P6 multiwell plates until complete adhesion and then incubated overnight in serum-free conditions. Cells were treated with human IL-36α or IL-1β (10 or 50 ng/ml).

### RNA isolation and real-time reverse transcription–polymerase chain reaction (RT-qPCR)

mRNA levels were determined using SYBR-green based quantitative PCR (qPCR). Briefly, RNA was extracted using a NucleoSpin kit according to the manufacturer’s instructions, and reverse-transcribed (RT) using a SABiosciences First Strand Kit. After the RT reaction, qPCR analysis was performed with a SABiosciences Master Mix and specific PCR primers for: human GAPDH (175 bp, PPH00150E, reference position 1287–1310, GenBank accession no. NM_002046.3); human IL-36 R (63 bp, PPH01077B, reference position 1073, GenBank accession no. NM_003854); human MMP13 (150 bp, PPH00121B, reference position 221-241, GenBank accession no. NM_002427.2); human NOS2 (132 bp, PPH00173E, reference position 3962, GenBank accession no. NM_000625.4); human COX-2 (63 bp, PPH01136F, reference position 1502, GenBank accession no. NM_000963.2). Amplification efficiencies were calculated for all primers utilizing serial dilutions of the pooled cDNA samples. The data were calculated, using the comparative (ΔΔCt) method and the MxPro software (Stratagene, CA, USA), as the ratio of each gene to the expression of the housekeeping gene. Data are shown as mean ± s.e.m (error bars) of at least three independent experiments and represented as fold-change vs. controls. Melting curves were generated to ensure a single gene-specific peak, and no-template controls were included for each run and each set of primers to control for unspecific amplifications.

### Western blot

Whole cell protein extraction was developed using a lysis buffer. Electrophoresis and blotting procedures have been described previously^13^. Immunoblots were incubated with the appropriate antibody (anti-phospho p38 diluted 1:1000, Millipore, MA, USA; anti-p38 diluted 1:1000, Millipore, MA, USA; anti IκBα diluted 1:1000, Cell Signaling Technology, MA, USA; anti-MMP-13 diluted 1:500, Santa Cruz Biotechnology, CA, USA; anti-NOS2 diluted 1:1000, Cell Signaling Technology, MA, USA; anti-COX-2 diluted 1:1000, Dako, Denmark) and visualized using an Immobilon Western kit (Millipore, MA, USA) and anti-rabbit (GE Healthcare, UK) horseradish-peroxidise-labelled secondary antibody diluted 1:2000. To confirm equal loading for each sample, after stripping in glycine buffer at pH3, membranes were reblotted with anti-β-actin antibody diluted 1:5000 (Sigma, MO, USA). Autoradiographs were analyzed with an EC3 imaging system (UVP, CA, USA).

### Immunohistochemical analysis (IHC)

Human articular cartilage samples were obtained with the permission of the local ethics committee from 10 OA patients and 10 healthy subjects. After fixation and paraffin embedding, sections of 4 μm were cut, deparaffinized in xylene and rehydrated through an ethanol series. Next, the sections were pre-treated with target retrieval solution high pH (Dako, Denmark). After that, we blocked endogenous peroxidase activity with peroxidase blocking reagent (Dako, Denmark) and incubated overnight with anti-IL-36α antibody (R&D Systems, MN, USA) and anti-goat secondary antibody (Dako, Denmark). For negative controls the appropriate secondary antibody was incubated in the absence of primary antibody.

For HRP, we used staining kit with DAB substrate (Dako,Denmark). The sections were counterstained with haematoxylin.

### Statistical analysis

Data are reported as mean ± S.E.M. (error bars) of at least three independent experiments. Statistical analyses were performed by unpaired *t*-test or One-way ANOVA followed by Bonferroni´s Multiple Comparison test, using the GraphPad Prism 4 software, with *p* values < 0.05 considered significant

## Results

### Expression of IL-36α in healthy and OA chondrocytes

**Figure 1:**
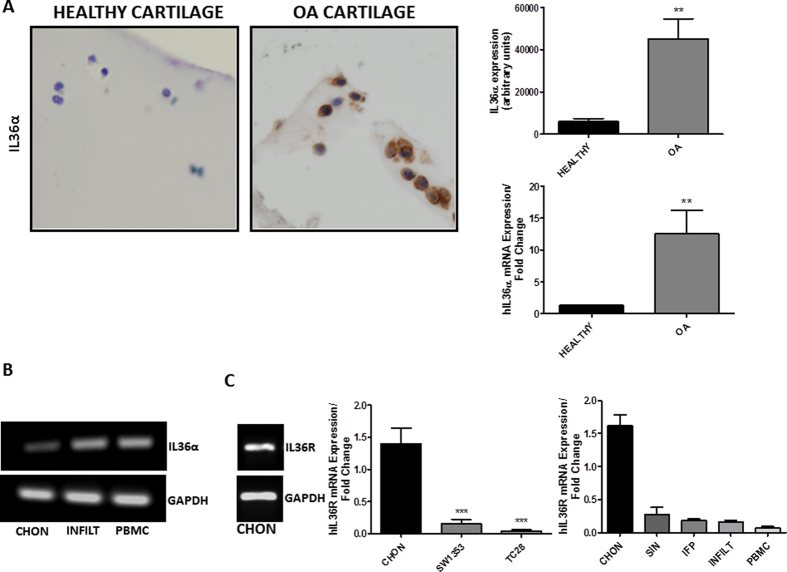
(**A**) Sections of knee articular cartilage from healthy (n = 10) and OA (n = 10) patients stained with anti-IL-36α (20X). Representative sections and the quantification of immunostaining are shown. IL-36α mRNA expression was also determined by qRT-PCR in human primary chondrocytes (**B,C**). Basal expression of IL-36α and IL-36R, evaluated by qRT-PCR in human primary chondrocytes, immune cells attached to cartilage, PBMCs, SW1353 cells, TC28 cells, synovial tissues and infrapatellar fat pad (n = 5). Amplicons were electrophoresed on 2% agarose gel, stained with ethidium bromide and visualized with a high definition CCD camera. Glyceraldehyde-3 phosphate dehydrogenase (GAPDH) expression is also shown. CHON = chondrocytes; INFILT = immune cells infiltrated into the joint; PBMC = peripheral blood mononuclear cells; SW1353 = SW1353 human chondrosarcoma cell line; TC28 = T/C-28a2 human chondrocyte cell line; SIN = synovial tissue; IFP = infrapatellar fat pad.

First, we aimed to analyze the expression of IL-36α in healthy and OA chondrocytes by IHC. As shown in Fig. 1A left panel, Immunostaining with IL-36 alpha antibody showed a barely low expression of IL-36α in healthy chondrocytes. However, OA chondrocytes likely expressed IL-36α more than healthy chondrocytes. This observation was confirmed through the quantification of the IHC and by the determination of IL-36α mRNA expression (Fig. 1A, right panel).

Migrated immune cells in the joint as well as PBMCs expressed higher levels of IL-36α than those observed in chondrocytes (Fig. 1B), suggesting that the immune cells of the joint could be a relevant source of this cytokine at joint level. Noteworthy, we detected high expression levels of the IL-36 R in human chondrocytes as compared with different human chondrocyte cell lines, joint tissues as well as immune cells (Fig. 1C).

### IL-36α induces MMP-13, NOS-2 and COX-2 in human chondrocytes

**Figure 2:**
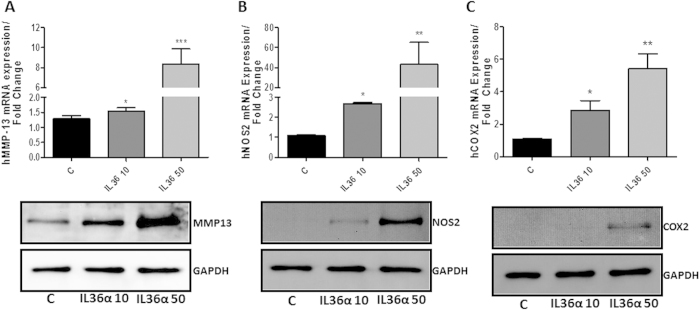
(**A–C**) Determination of human MMP-13, NOS2 and COX-2 mRNA and protein expression by qRT-PCR and western blot respectively after 24 hours recombinant IL-36α treatment in human primary chondrocytes. The results shown were obtained of at least three independent experiments, using at least three OA articular chondrocytes independent cultures.

We next sought to characterize some biological responses of human recombinant IL-36α by assessing the effect on the induction of certain well-known factors that contribute to cartilage degradation. As shown in Fig. 2A–C, recombinant IL-36α induced a significant increase in mRNA expression of MMP-13, NOS-2 and COX-2 in human primary cultured chondrocytes. The increase was dose dependent and these results were also confirmed in terms of protein expression (Fig. 2A–C low panels).

### Comparative effect of IL-36α and IL-1β on the induction of MMP-13, NOS2 and COX-2

**Figure 3:**
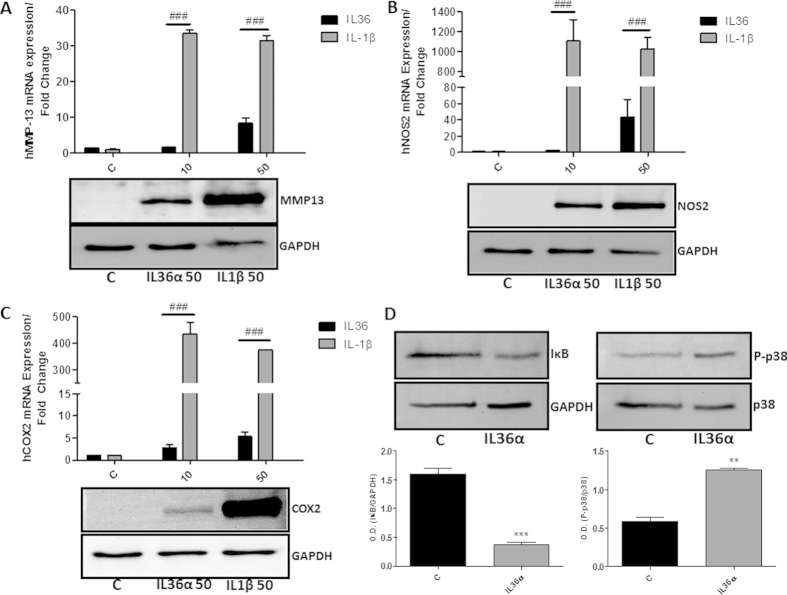
(**A–C**) Determination of human MMP-13, NOS2 and COX-2 mRNA and protein expression by qRT-PCR and western blot respectively after 24 hours recombinant IL-36α or IL-1β treatment in human primary chondrocytes. (**D**) Determination of the degradation of IκB and the phosphorylation of p38 by western blot. β-actin and total p38 were used to confirm equal load. The results shown were obtained of at least three independent experiments, using at least three OA articular chondrocytes independent cultures. **Low panels.** Data showing densitometric analysis of all performed western blots.

We analyzed the activity of IL-36α in comparison to other members of the IL-1 family, as IL-1β. As shown in Fig. 3A–C, IL-36α seems to be less efficient than IL-1β in inducing MMP-13, NOS2 and COX-2.

### IL-36α activates p38 and NFκB pathway

As described above, chondrocytes express IL-36R. Therefore, we wanted to obtain more details into the intracellular signaling pathways used by IL-36α. For this purpose we tested whether IL-36α activity occurred through NFκB/p38 signaling pathway in chondrocytes. As shown in Fig. 3D, IL-36α was able to induce a significant degradation of the inhibitory protein IκB in human primary chondrocytes. IL-36α was also able to increase the phosphorylation of p38 in the same cell type (Fig. 3D).

## Discussion

Recently, IL-36α has been identified as a new member of the IL-1 cytokine family with supposed pro-inflammatory properties. IL-36α seems to be a relevant factor involved in the development of psoriasis^9^. Moreover, it has been postulated that IL-36α might mediate the inflammatory response in RA and PsA synovial tissues^10^. However, scarce or null information about the role of IL-36α at cartilage level is available.

To understand the cellular and molecular events responsible of putative IL-36α activity in cartilage, we first sought to analyze IL-36α expression in healthy and OA cartilage, next we evaluated chondrocyte response to exogenous IL-36α administration *in vitro*.

In our study we show for the first time the comparative expression of IL-36α in healthy cartilage versus OA cartilage. The enhanced expression of IL-36α found in OA chondrocytes, when compared to healthy chondrocytes, suggested that, as observed for other cytokines such as IL-1^2^,^14^, the production of IL-36α by articular cartilage was altered during OA. In addition, our data showed that immune cells infiltrated into the joint of OA patients can be considered as relevant IL-36α-expressing cells intraarticularly. This aspect, together with the observation that chondrocytes, as well as circulating PBMCs showed high levels of expression of IL-36α suggested that these cells might contribute to the progression and/or perpetuation of the inflammatory response at joint level. In addition, it was reported that in the context of inflammatory arthritis, IL-36α was predominantly produced by synovial plasma cells, triggering thereafter an inflammatory response in synovial fibroblasts^10^, these results suggested that IL-36α could be a link between adaptive immunity and inflammatory response in different pathologies such as RA or PsA. Also, in skin inflammatory diseases, IL-36 has been demonstrated to be crucial for the regulation of the immune response. IL-36R knockout mice presented much less neutrophils and macrophages infiltration into the skin lesions as well as decreased T cell expansion in comparison to wild type mice after the induction of psoriasiform dermatitis^15^. In line with this, transgenic mice overexpressing IL-36α also showed an increased immune cell infiltrate in the dermis, which is consistent predominantly by macrophages, neutrophils and lymphocytes^9^.

Our results showed also that IL-36α induced efficiently inflammatory mediators such as NOS2 and COX-2. These results are in agreement with those published by Magne *et al.* showing that treatment with the β isoform of IL-36 enhanced the expression of different inflammatory mediators such as IL-6 or nitric oxide in chondrocytes^11^. As far as we are aware, this is the first clear experimental evidence of the induction of NOS2 and COX-2 by IL-36α in human cultured OA chondrocytes.

Another relevant and novel data showed in our study is that IL-36α was able to up-regulate the expression of MMP-13, one of the most important collagenases involved in OA^16^. MMP-13 is induced by other members of the IL-1 cytokine family members, such as IL-1^17^,^18^. Many other cytokines of this family, after binding to their own receptors, can activate catabolic pathways in normal and OA chondrocytes through the release of MMPs^19^ and the suppression of proteoglycan synthesis^2^.

However, this is the first evidence of a direct and strong up-regulation of MMP-13 expression after IL-36α stimulation in chondrocytes. Taken together, these results suggest that IL-36α elicited a clear detrimental effect by increasing the expression of enzymes able to promote cartilage breakdown in OA.

In our study we observed that IL-36α was less potent than a classic pro-inflammatory cytokine, IL-1β. Actually, chondrocytes treated with IL-1β, at the same doses of IL-36α, showed a higher induction of the target-analyzed genes. Our observation is in agreement with the fact that lower amounts of IL-1β are needed to activate the NFκB signaling pathway in comparison to IL-36α or IL-36β^6^. We determined that the activity of IL-36α seems to be higher than the isoform IL-36β. Actually, different studies demonstrated that the activity of IL-36β, as pro-inflammatory agent, became significant at doses above 100 ng/mL^6^,^11^. In our experimental set, we observed that IL-36α exerted a significant effect at lower doses, 10 or 50 ng/mL in cultured chondrocytes.

For completeness, here we show that human chondrocytes express IL-36R mRNA efficiently, even more than other joint tissues. We also demonstrate that cell stimulation with recombinant IL-36α, likely through IL-36R, activates specific MAPK/NFκB signaling pathway. These effects are in agreement with those observed with other members of the IL-1 cytokine family^17^,^20^.

In our experimental conditions, we observed a rapid degradation of IκB in human cultured chondrocytes. The degradation of IκB, the inhibitory protein joined to NFκB, is necessary for NFκB translocation to the nucleus. A similar observation was obtained in IL-36α –challenged synovial fibroblasts^10^.

Our findings are also in agreement with previous observations showing that p38 phosphorylation is an important step in the NFκB pathway. Indeed, although the activation of NFκB and that of p38 MAPK are mediated by different pathways, both may converge. This mechanism is strongly supported by experimental evidence with other cytokines that demonstrate the activation of p38 in human OA chondrocytes, which in turn activates the MAPKAP and trans activates NFκB^21^.

**Figure 4:**
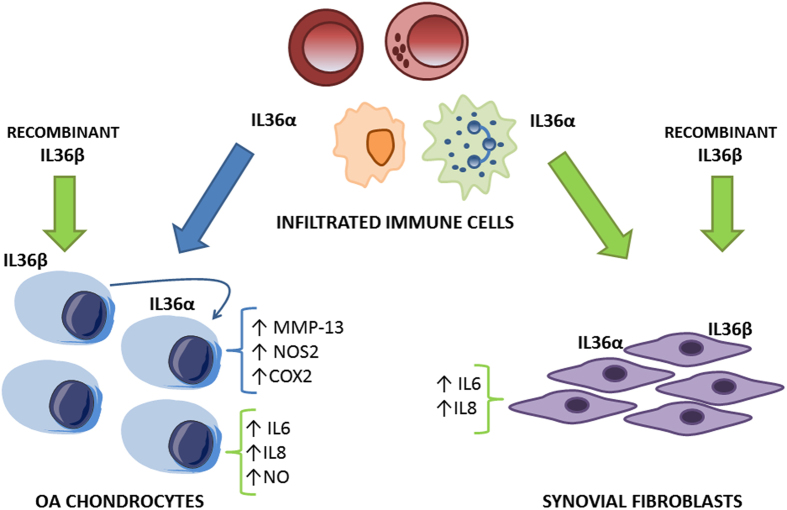
Illustrated summary. Blue arrows and blue keys represent results obtained in the present study. Green arrows and green keys represent results obtained in other published articles^10^,^11^. IL36α produced by immune cells could induce the expression of different pro-inflammatory and pro-catabolic factors in chondrocytes and in synovial fibroblasts. Probably, IL36α also acts in an autocrine or paracrine manner in chondrocytes. Moreover, it was reported that the addition of recombinant IL36β to chondrocytes or synovial fibroblasts was able to induce the expression of different pro-inflammatory mediators.

In conclusion, in the present study we demonstrated that chondrocytes express IL-36α and, for the first time, that this cytokine is able to induce the expression of the collagenase MMP-13, as well as other pro-inflammatory factors, including NOS2 and COX-2. All together, these molecules can cooperate with one another resulting in enhancement and perpetuation of the matrix degrading processes at cartilage level. IL-36α also activates p38 and NFκB signaling pathways in chondrocytes, suggesting that the classic route triggered by proteosomal degradation of IκB to release NFκB complex is at play. Our results, together with other reports, demonstrated that IL-36α induces catabolic mediators and the synthesis of pro-inflammatory cytokines and chemokines (Fig. 4). However, further studies on the role of this novel cytokine are needed to completely assess the role of IL-36α in the pathogenesis and progression of osteoarthritis.

## Additional Information

**How to cite this article**: Conde, J. *et al.* IL-36α: a novel cytokine involved in the catabolic and inflammatory response in chondrocytes. *Sci. Rep.* **5**, 16674; doi: 10.1038/srep16674 (2015).
